# 

**Published:** 1892-07-23

**Authors:** 


### CONTENTS.

**Mr. Gladstone and the Medical Profession** - - - - 273

**Passing Topics.**—Putting into Practice - - - - - 274

**Medical History from the Earliest Times.** By E. T. Withington, M.A., M.B.Oxon. XVIII.—The Methodic School 275

**Lunacy in Scotland** - - - - - - - - - 276

**The Attitude of Great Writers towards Disease.** VI.—Robert Burton - - - - - - - - - - 277

**Everybody's Page** - - - - - - - - - - - 278

**Annotations.**—Flowers and Flower Girls—Election Fever—A Lifeboat Saturday - - - - - - - - - - 279

**Notes and News** - - - - - - - - - - - 280

**Around the Hospitals—Scraps and Gleanings** - - - 281

**The Practitioners' Mirror and Retrospect—**

Medicine.—Thyroid Extract for Myxœdema. II. - - - 282

Surgery.—The Operative Treatment of Empyena and Abscess of the Lung—Some Conditions Simulating Renal Calculus - 282

Therapeutics.—Phenocoll Hydrochlorate - - - - 284

**The Institutional Workshop—**

Hospital Administration. III.—Finance - - - - 286

Within the Hospitals. VII.—Royal South Hants Infirmary, Southampton - - - - - - - - - - - 285

Hospital Construction. The New Hospital, Aden - - 287

Practitioners' Bookshelf.—Hygiene and Sanitary Science - 287

**The Multiplication of Metropolitan Hospitals** - - 288

### EXTRA SUPPLEMENT.—THE NURSING MIRROR.

**En Passant.**—Registration of Nurses—Warwick Nursing Association—The Home of Comfort for Epileptics—Nurses Co-operation—*Truth* and the "London"—The London Hospital Examination—Tea and Talk—Short Items—Uniform—Three Years' Training - - - - - - - - - - cxiii

**Ventilation, Disinfection, and Diet.** By P. Caldwell Smith. XV.—Diet and Dietaries—(*continued*) - - - - cxiv

**Staff Nurses** - - - - - - - - - - - cxv

**Hints on Hands** - - - - - - - - - - cxv

**Notes from Australia** - - - - - - - - - cxvii

**Presentation** - - - - - - - - - - - - cxvii

**A Plan of Instruction in Invalid Cooking for Training Schools.** By Isabel A. Hampton, Superintendent of Nurses, and Principal of the Training School of the Johns Hopkins Hospital - - - - - - - - - - cxvi

**Wants and Workers** - - - - - - - - - cxvii

**Notes and Queries** - - - - - - - - - cxvii

**Everybody's Opinion.**—Holidays in Switzerland—The Matronship of Longton Cottage Hospital—Hospital and Asylum Nurses—One Who Wants to Know - - - - - - cxviii

**For Reading to the Sick** - - - - - - - - cxix

**Off Duty.**—One Touch of Nature - - - - - - - cxix

